# Aging of Industrial Polypropylene Surfaces in Detergent Solution and Its Consequences for Biofilm Formation

**DOI:** 10.3390/polym15051247

**Published:** 2023-02-28

**Authors:** Julian Cremer, Bernhard P. Kaltschmidt, Annika Kiel, Jens Eberhard, Stephan Schmidt, Christian Kaltschmidt, Barbara Kaltschmidt, Andreas Hütten, Dario Anselmetti

**Affiliations:** 1 Department of Experimental Biophysics & Applied Nanoscience, Faculty of Physics, Bielefeld University, 33615 Bielefeld, Germany; 2 Department of Thin Films and Physics of Nanostructures, Center of Spinelectronic Materials and Devices, Faculty of Physics, Bielefeld University, 33615 Bielefeld, Germany; 3 Department of Cell Biology, Faculty of Biology, Bielefeld University, 33615 Bielefeld, Germany; 4 Miele & Cie. KG, 33611 Bielefeld, Germany

**Keywords:** polypropylene, liquid aging, detergent, filler, ethylene bis stearamide, biofilm, *Pseudomonas aeruginosa*

## Abstract

The performance of plastic components in water-bearing parts of industrial and household appliances, often in the presence of harsh environments and elevated temperatures, critically relies on the mechanical and thermal polymer stability. In this light, the precise knowledge of aging properties of polymers formulated with dedicated antiaging additive packages as well as various fillers is crucial for long-time device warranty. We investigated and analysed the time-dependent, polymer-liquid interface aging of different industrial performance polypropylene samples in aqueous detergent solution at high temperatures (95 °C). Special emphasis was put on the disadvantageous process of consecutive biofilm formation that often follows surface transformation and degradation. Atomic force microscopy, scanning electron microscopy, and infrared spectroscopy were used to monitor and analyse the surface aging process. Additionally, bacterial adhesion and biofilm formation was characterised by colony forming unit assays. One of the key findings is the observation of crystalline, fibre-like growth of ethylene bis stearamide (EBS) on the surface during the aging process. EBS is a widely used process aid and lubricant enabling the proper demoulding of injection moulding plastic parts. The aging-induced surface-covering EBS layers changed the surface morphology and promoted bacterial adhesion as well as biofilm formation of *Pseudomonas aeruginosa*.

## 1. Introduction

In general, it is assumed that plastic lasts forever. It is true that plastic decomposes very slowly, but for several applications the actual service life of plastic parts, satisfying a certain requirement, is short. In order to keep the plastic’s functionality and prevent it from early replacement, the study of polymer aging is indispensable. To improve the durability of polymers, additives are essential to protect them against harmful conditions [1]. For instance, UV-light stabilisers are widely used and studied, but for many applications antioxidant agents are more necessary [2,3,4,5,6]. Aside from the stabilisation of a polymer, the processability and other functional properties are addressed with additives too, using for instance lubricants, matting, or antistatic agents [1,7]. Moreover, additives can be used to obtain antibacterial properties [8]. Since additives typically have a low molecular weight and their solubility in a polymer can be low, blooming often occurs, changing the surface morphology and chemistry [9]. While longevity in terms of sustainability is an advantage on the one hand, it is a cause for concern with regard to microbial contamination, especially for household appliances [10,11,12,13]. Sterility is the top priority for many applications, no matter how good the processability or durability might be.

Polypropylene is a semi-crystalline thermoplastic polymer with remarkable chemical and temperature resistance. Therefore, it is used for numerous applications in the automotive, household, food, or medical industry [14,15]. The surface properties are, among other things, determined by the manufacturing process [16]. For example, injection moulding as a fast manufacturing-process is especially suited for large numbers of identical parts. In 2020, nearly 25.7 million tons of polypropylene was processed by injection moulding [17]. Associated with this manufacturing process is the skin-core morphology of these plastic parts. The skin layers are more structured and oriented than the core. Due to the shear forces acting at the walls of the casting mould, the molecular chains can be forced to align in the injection direction [18,19,20].

In this paper, the aging process of three different industrially used polypropylene homopolymer samples in an oxidative detergent solution at 95 °C is presented. Contrary to thermal oxidation or aging induced by UV radiation, aging in oxidative liquids is rarely studied. The polypropylene samples were examined after 10 different time steps up to 4000 h (nearly 24 weeks). One sample contains 40% of talc (PP-T), one 30% of glass fibre (PP-G), and one is unfilled (PP). Talc and glass fibres are one of the most used fillers in the market and are mainly inserted for reinforcement. All samples were produced by injection moulding and contain additive packages. The material degradation and additive blooming was recorded using scanning electron microscopy (SEM) and atomic force microscopy (AFM). Attenuated total reflection Fourier transformed infrared spectroscopy (ATR-FTIR) was used to identify the bloomed processing additive as ethylene bis stearamide (EBS). To the best of our knowledge, the here reported severe extent of EBS crystal growth on the surface has not been studied or observed before. Additionally, differential scanning calorimetry (DSC) measurements were performed to provide knowledge about the crystallinity and average melting point of the samples.

To study the EBS influence on a biological level, colony-forming units of the biofilm producing bacteria *Pseudomonas aeruginosa* were recorded on polypropylene samples with and without EBS on the surface. The influence of aged polypropylene on bacterial adhesion and biofilm production was also investigated. To gain insights into the effect of EBS itself on biofilm formation, pure EBS in powder form was used for a microtiter plate assay. A wild-type *Pseudomonas aeruginosa* strain isolated from household appliances was used for these experiments [13].

## 2. Materials and Methods

### 2.1. Polypropylene Samples and Aging Process

#### 2.1.1. Injection-Moulded Polypropylene Samples

Multi-purpose tensile specimens were manufactured according to ISO 294-1 (polymer mass temperature 240 °C, 250 °C for PP-G, mould temperature 40 °C for all grades). The polypropylene tensile specimens have a length of 105 mm, a width of 20 mm and 10 mm, respectively, and a thickness of 0.8 mm (engineering drawing Figure S2).

Sample PP contains no filler, the average molecular weight (Mw) is 350,000 g/mol (Mn 76,000 g/mol, polydispersity (PD) 4.6). Sample PP-T contains 40% of talc and the base polymer has a Mw of 242,000 g/mol (Mn 48,700 g/mol, PD 4.97). Sample PP-G contains 30% glass fibres while the base polymer has a Mw of 240,000 g/mol (Mn 58,000 g/mol, PD 4.14). All grades were designated for use in the household appliance and white good industry according to the datasheet and therefore we derived that the grades are accordingly stabilized while the exact combination and amount of the additives remains unknown to us due to industrial confidentiality of the polymer manufacturer. Qualitatively identified where phenolic antioxidants AO1010 (CAS 6683-19-8 for PP-T) and AO1330 (CAS 1709-70-2 for PP and PP-G) as well as phosphite antioxidant AO168 (CAS 31570-04-4 for PP, PP-G and PP-T).

#### 2.1.2. Aging Process

Aging of the test specimen was conducted via a SPS-automated media-storage equipment with temperature control (95 ± 2 °C), stirring (150 rpm), and continuous dosage and fill level control to ensure a consistent concentration of active detergent solution over time. Detergent concentration was 8 g/L. Fleet ratio >350:1 (ratio of the volume from 350 parts detergent to 1 part polypropylene sample). Samples were withdrawn manually at given time points, let cool and flushed once with DI water and isopropanol.

The main ingredients of the detergent in accordance with EU regulation 648/2004 on detergents of the used all-purpose test detergent were as follows: sodium dodecylbenzenesulfonate (5–10%, CAS 68411-30-3), sodium laureth sulphate (1.25–2.5%, CAS 68411-30-3), oxygen-based bleaching agent (Hydrogenperoxide) (2.5–5%, CAS 7722-84-1), sodium borate (0.5–1.25%, CAS 1330-43-4), and sodium palm kernelate (CAS 61789-89-7). It should be noted that not every detergent contains oxygen-based bleaching agents, such as colour detergents.

### 2.2. Physical Methods

#### 2.2.1. Scanning Electron Microscopy (SEM)

The used SEM was a Helios NanoLab DualBeam 600 (FEI Company, Hillsboro, OR, USA) scanning electron microscope. Prior to analysis, the samples were sputter coated with a layer of 4 nm ruthenium and connected to the stage with conducting tape to ensure proper conduction. The measurements were carried out at an acceleration voltage of 5 kV with a beam current of 0.17 nA.

#### 2.2.2. Atomic Force Microscopy (AFM)

The AFM measurements were conducted with a NanoWizard™ Ultra Speed 2 (JPK-Bruker, Berlin, Germany). The data were recorded using a Tap300Al-G cantilever from Budget Sensors^®^ (Innovative Solution Bulgaria Ltd., Sofia, Bulgaria) operating in tapping mode. The data were evaluated with the software provided by the AFM manufacturer.

#### 2.2.3. ATR-FTIR

FTIR measurements were performed with a Vertex 70 (Bruker, Billerica, MA, USA) using the MIRacle™ ATR accessory (Pike technologies, Madison, WI, USA) with a 3-reflection diamond/ZnSe crystal plate and a 6 mm aperture. Fifty scans per sample were recorded with a resolution of 2 cm^−1^ in the range of 600–4000 cm^−1^. An automatic baseline correction was carried out by the measurement software. Pure polypropylene (Mw 340,000 g/mol and Mn 97,000 g/mol) (CAS 9003-07-0) and ethylene bis stearamide (EBS) (CAS 110-30-5) were purchased from Sigma-Aldrich and abcr GmbH & Co. KG, respectively.

#### 2.2.4. DSC

DSC measurements were carried out by a DSC 25 (TA Instruments, Waters Corporation, Milford, Massachusetts). A total of 3 mg of each sample were used for measurement and the measured temperature range was −30 to 200 °C, with a scanning speed of 10 °C/min. A triplicate (3 samples) per timestep was measured. The enthalpy accuracy is ±0.1% according to the manufacturer’s specification. The DSC was calibrated with Indium. The crystallinity of the PP samples was determined by integrating over the melting point peak of the heating curve to calculate the enthalpy, as published by R. Blaine and recommended by the manufacturer (TA Instruments) [21].

### 2.3. Biological Methods

#### 2.3.1. Bacterial Strains

The biofilm producing strain *Pseudomonas aeruginosa*, isolated from a domestic washing machine, was chosen for all experiments [13]. The accession number of the *P. aeruginosa* strain is PRJNA838002. The bacterial isolate was inoculated from frozen stocks stored at −80 °C and cultured on LB agar plates for 24 h prior to experimental use.

#### 2.3.2. SEM Investigation of Attached Cells and Biofilm Formation

For an ultrastructural observation of biofilms grown on unaged and aged polypropylene samples, the samples were cut into 1 × 1 cm pieces. Since the surface sides differ slightly, we tried to use the same side for all experiments. PP samples were placed into a 6 well plate. Wild-type *P. aeruginosa* isolate was inoculated from fresh LB agar plates and pre-cultured in 10 mL LB medium at 37 °C overnight under shaking conditions. For each sample, 2 mL bacterial suspension (in LB-medium), adjusted at OD600 = 0.1, was filled into the well. The samples were incubated for 24 h at 37 °C under static conditions. After the incubation period, planktonic cells were washed away by submerging the samples two times in physiological saline (0.9% NaCl) followed by one time in bidest H_2_O. The samples were fixed with half-strength Karnovsky’s solution (2% paraformaldehyde, 2.5% glutaraldehyde) for 30 min. The fixed samples were dehydrated using 50, 70, 80, 90, 95, and 100% (*v*/*v*) graded ethanol. To improve the conductivity of the samples, they were sputter-coated with a layer of 4 nm ruthenium.

For an observation of pure ethylene bis stearamide (EBS), C_38_H_76_N_2_O_2_ (CAS 110-30-5) in micro-powder form (abcr GmbH & Co. KG, Karlsruhe, Germany) was mixed with bidest H_2_O at a concentration of 1 g/L. A droplet of 25 µL was placed onto a sterile glass slide, with a size of 22 × 22 mm, and dried under a laminar flow bench. A size measurement of the diameter of the observed particles was performed by using the open-source FIJI (ImageJ) software.

For an observation of the influence of pure EBS on biofilm formation, glass slides were placed into a 6 well plate. LB medium was supplemented with 1 g/L EBS powder. A total of 2 mL medium, inoculated with *P. aeruginosa* and adjusted at OD600 = 0.1, was filled into the well plates and incubated for 24 and 48 h. After the two incubation periods samples were washed and fixated as described before.

#### 2.3.3. Colony Forming Unit Assay (CFU)

PP samples were incubated in LB-medium inoculated with *P. aeruginosa* as described. As a reference material, sterile glass slides, with a size of 22 × 22 mm were used. Three samples of each material were tested. After an incubation period of 24 h, planktonic cells were washed away by submerging the samples three times in physiological saline (0.9% NaCl). The biofilm/attached cells were detached from the surface by vigorous vortexing for at least 1 min (Vortex Genie, Fisher Scientific, USA) in 10 mL physiological saline (0.9% NaCl). To verify whether the biofilm has completely detached from the surface, we used the SEM to scan the surface of the vortexed samples for remaining bacteria. No bacteria were present on the surface, only residues that had formed due to previous adhesion (Figure S4). A serial dilution series was performed, and 100 µL of each dilution was plated onto fresh LB agar plates. The plates were incubated overnight at 37 °C. After incubation, colonies were counted and the colony-forming units per mm^2^ were calculated.

#### 2.3.4. Microtiter Plate Assay (MTP)

The quantitative microtiter plate assay as described by Christensen et al. was performed [22]. *P. aeruginosa* was inoculated from fresh agar plates and pre-cultured in 10 mL LB medium at 37 °C overnight under shaking conditions. LB full medium and MOPS minimal medium, described by Neidhardt et al. and MOPS minimal medium supplemented with 1% glucose, was used [23]. All media were supplemented with EBS powder in different concentrations [g/L]: 10, 5, 1, 0.1, and 0.01. Pre-cultures with P. aeruguninsa were inoculated of all concentrations and adjusted to OD600 = 0.01. A total of 96 flat bottom well plates (SARSTEDT AG & Co. KG, Nümbrecht, Germany) were used for the experiment. For each concentration 6 wells were filled with 200 µL media inoculated with *P. aeruginosa* (6 technical replicates). Due to the frequent temperature induced edge effect, the outer wells were not used. Plates were incubated for 24 and 48 h at 37 °C under static conditions. After incubation the planktonic cells were washed away by filling the wells three times with 200 µL bidest H_2_O. Afterwards plates were air dried for 24 h. Remaining biofilm residues were stained with 0.1% Crystal violet (CV) for 30 min. Crystal violet was discarded and wells were washed three times with bidest H_2_O. Remaining stained biofilm was dissolved with 225 µL of 30% acetic acid. A 10-fold dilution was performed in a fresh 96 flat bottom well plate with an end volume of 200 µL. Optical densities were spectrophotometrically measured at 590 nm using a PowerWave microplate reader (BioTek, Winooski, VT, USA). Control plates without bacteria were conducted to ensure that the observed staining effects were not due to EBS residues. Plates were incubated, washed, and stained in the same manner. The values obtained for the optical density were then subtracted from the values obtained in the presence of *P. aeruginosa*.

## 3. Results

### 3.1. Microscopy

**Figure 1:**
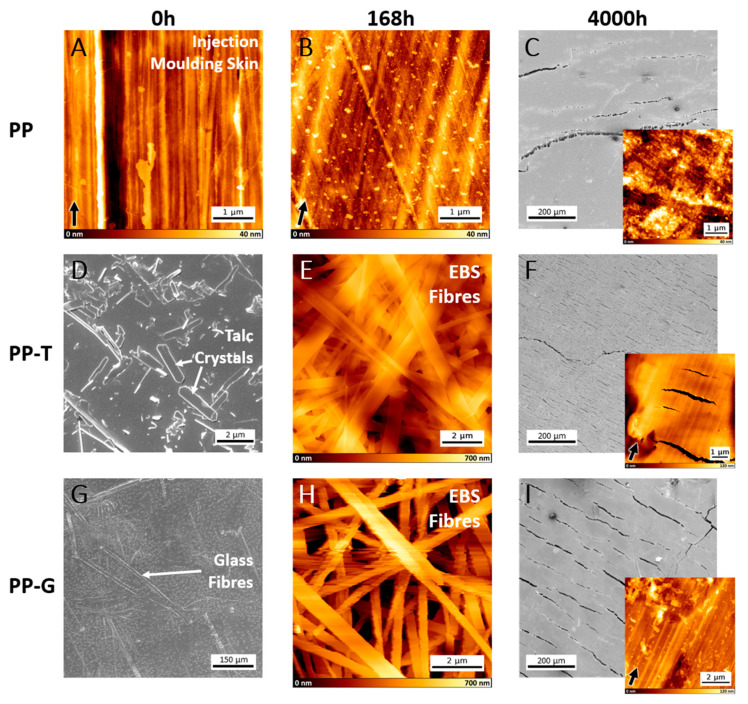
SEM images in grey and AFM images in gold-brown show the structural changes during aging of samples PP, PP-T, and PP-G. The first column (**A**,**D**,**G**) depicts the untreated state of the samples with the typical injection moulding surface and the filling material. Two samples show fibre-like EBS on the surface after 168 h in the detergent at 95° (**E**,**H**). After 4000 h the crack formation (**C**,**F**,**I**) as well as the difference in degradation of the skin layer (**C**,**F**,**I** inlay) can be seen. Black arrows indicate the injection direction, white arrows the fillers.

The injection moulding process predominantly determines the surface structure of the polypropylene samples. AFM was deployed to record the changes in the injections moulding skin during aging. Since SEM is better suited to scan large areas fast, it was used to image the filling material and follow the aging on a bigger scale. Figure 1 shows AFM and SEM images of the three polypropylene samples for three different timesteps of detergent treatment at 95 °C (0 h, 168 h, and 4000 h). The AFM images have a gold-brown false colour height ruler, and the SEM images are depicted in grey. The first row shows the unfilled polypropylene sample (PP). The typical surface structure of all untreated polypropylene samples can be seen in the first image (Figure 1A). The injection moulding direction is indicated by the arrow in the lower left corner of the images. The surface exhibits parallel grooves which correspond to the direction the polypropylene was injected into the mould. AFM images of all three unaged samples are shown in the Supplementary Materials (Figure S3). They are not shown here because the surface of the unaged samples does not differ except for the fillers. The injection moulding skin is still clearly visible on the PP sample after 168 h, only small aggregations on the surface can be detected, most likely due to additive blooming (Figure 1B). After 2000 h (~12 weeks) this skin structure is still intact, meaning the material removal rate on the surface is very low in general (not shown here). The initial skin layer is removed after 4000 h (~24 weeks) (Figure 1C AFM). Additionally, deep cracks appeared after this time, dictating the end of the service life for thin-walled parts (Figure 1C SEM). Presenting only minute changes in the surface topography up to 4000 h, the PP sample demonstrates a high stability in a heated oxidative environment.

The SEM and AFM analysis of sample PP-T, filled with 40% talc, is depicted in the second row of Figure 1. Besides the typical polypropylene skin layer, the untreated sample has some talc crystals on the surface (Figure 1D white arrows). After 168 h in the detergent solution at 95 °C the surface is nearly completely covered by a fibre-like layer (Figure 1E). The crystalline fibres first bloom and then grow on the surface due to the heated liquid environment. The layer thickness could be measured by AFM and reached about 2 µm (Figure S1). These fibre-like crystals consist of ethylene bis stearamide (see IR-spectroscopy part). After 168 h, a massive number of fibre-like crystals have formed on the surface, and as time passes, they begin to detach and dissolve from the surface. After 3000 h in the detergent solution, the EBS is only left in remnants on the surface. Cracking already starts after 2000 h and reaches a severe extent after 4000 h (Figure 1F). Noteworthy is the shape of the cracks compared to the other samples. Here many small cracks spread on the surface whereas the other samples show far less but larger and deeper cracks. We argue that this occurs on account of the talc filling. With many hard and brittle talc crystals in the polymer matrix, the cracks always stop reaching such a talc crystal, forcing the strain release to develop in the form of many small cracks.

Sample PP-G with 30% glass fibres is shown in Figure 1G (white arrows). This sample exhibited the same fibre-like EBS growth on the surface as the PP-T sample after 168 h (Figure 1H). Here the EBS remained on the surface longer, as many EBS fragments could still be detected after 4000 h (Figure 1I AFM). Only after 4000 h cracks appeared, like for sample PP (Figure 1I SEM).

Samples PP-T and PP-G still possessed the injection moulding skin structure after 4000 h. Comparing the 4000 h inlaid AFM images, it is obvious that the PP sample lost the initial surface structure meaning the parallel align grooves are gone, whereas these grooves are still present for PP-T and PP-G after this time. Therefore, the EBS fibre-like network on the surface of PP-T and PP-G may act as a temporary sacrificial layer, preventing the polypropylene surface from direct contact to the oxidative solution.

### 3.2. ATR-FTIR Spectroscopy

**Figure 2:**
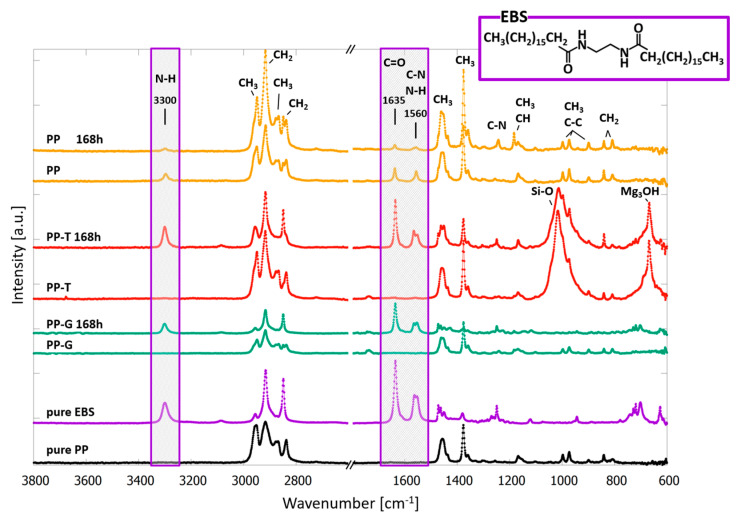
ATR-FTIR spectra of pure PP, pure EBS, PP-G after 0 h and 168 h, PP-T after 0 h and 168 h, and PP after 0 h and 168 h in the detergent at 95 °C. EBS bands 3300, 1635, and 1560 cm^−1^ are highlighted. The EBS structural formula is presented in the upper right corner.

ATR-FTIR measurements were performed to obtain a chemical fingerprint of the surfaces. The penetration depth of the infrared light with the used ATR ZnSe crystal is less than 2 µm, providing a surface sensitive measurement, ideal to identify bloomed additives [24]. The samples were measured in the untreated initial state and after every timestep of detergent treatment. Here the spectroscopy data were used to identify the grown fibre-like crystals on PP-T and PP-G as ethylene bis stearamide (EBS). EBS exhibits dominant IR absorption at 3300, 1635, and 1560 cm^−1^. The spectra have been recorded for pure EBS (purple curve) and pure polypropylene with no additives or fillers (black curve) and were compared to the untreated and aged samples in detergent solution (Figure 2). For the untreated sample PP, the EBS could be detected on the surface and the amount on the surface decreases with increasing time in the detergent solution (orange curves). In contrast, no EBS absorption bands could be detected on the surface of the untreated samples PP-T and PP-G (red and green curves). The EBS bands appeared only after exposure to the detergent solution and are most dominant for the 168 h samples corresponding to the massive fibre-like EBS growth on the surface detected with AFM and SEM. We can exclude the presence of the often-used lubricant erucamide due to the lack of NH_2_ bands at 3395 and 3183 cm^−1^ in the spectra. Blooming of phenolic antioxidants can be excluded as well because there are no phenolic absorptions bands around 3600 cm^−1^ measurable. The talc in sample PP-T can be detected in the FTIR spectra by the Si-O and Mg_3_-OH bands at 1015 and 675 cm^−1^, respectively. The ATR crystal plate itself shows around 2200 cm^−1^ several absorption bands. Since these bands do not originate from the samples, they are not shown here to enable a better visualisation of the data.

### 3.3. DSC

**Figure 3:**
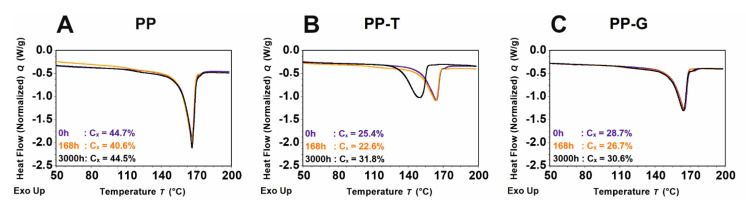
DSC graphs of samples (**A**) PP, (**B**) PP-T and (**C**) PP-G after 0 h, 168 h and 3000 h, respectively, with corresponding crystallinity. Minimum of the heat flow corresponds to the melting temperature.

Additional information about the material properties, crystallinity, and average melting point of the samples were obtained by DSC measurements (Figure 3). Here the three different timesteps 0 h, 168 h, and 3000 h were chosen. The 3000 h timestep was selected over the 4000 h timestep to compare the already cracked PP-T with the still intact PP and PP-G. A value of 207 J/g was chosen as the reference for 100% crystalline isotactic polypropylene [25]. The PP sample shows the highest crystallinity of the three samples with values between 40.6 to 44.7%. The melting point of sample PP stays nearly constant for all measured timesteps with 166.5 °C.

The DSC analysis of PP-T revealed a significant change after 3000 h of aging compared to the unaged sample. The endothermic peak of the 3000 h sample is shifted to the left which results in a drop of the melting temperature from unaged T_0h_ = 164.3 °C to 3000 h aged T_3000h_ = 150.4 °C. This is a sign of chain scission because shorter polymer chains result in a lower melting point. In contrast the crystallinity increases from 25.4% to 31.8%. This increase in crystallinity could be related to the fact that lower molecular weight polymers tend to crystallize more easily and facilitate secondary crystallization [26]. The difference between the sample after 168 h of aging and the unaged sample is in comparison much smaller, with a slightly lower melting temperature of T_168h_ = 163.8 °C and a lower crystallinity of 22.6%.

The PP-G sample shows relatively consistent DSC graphs in relation to the melting temperature. The melting temperature drops only from T_0h_ = 165.2 °C to T_3000h_ = 164.4 °C. The crystallinity on the other hand drops from unaged 28.7% to 26.7% for the 168 h aged sample and then rises to 30.6% for the 3000 h aged one. The increase in the crystallinity could also be related to secondary crystallization as seen in the PP-T sample.

The DSC measurements reveal no significant aging signs for the PP and PP-G samples. This is in agreement with the microscopy results showing no crack formation on the surface after 3000 h of aging. The PP-T sample on the other hand indicates with the peak shift a serious molecular degradation.

### 3.4. Bacterial Cultivation/CFUs

**Figure 4:**
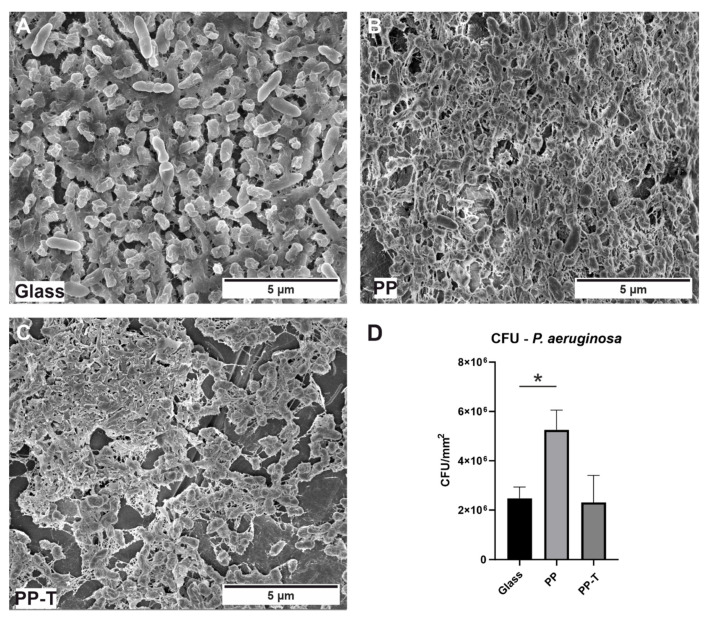
Bacterial attachment on the unaged as received polypropylene samples. SEM image of (**A**) *P. aeruginosa* attached to a reference glass sample, (**B**) unfilled PP, and (**C**) talc filled PP-T. (**D**) Quantification of the attached *P. aeruginosa* onto the surfaces by CFU Assay. Mean ± standard error of the mean was statistically analysed by Mann–Whitney U tests (* *p* ≤ 0.05).

**Figure 5:**
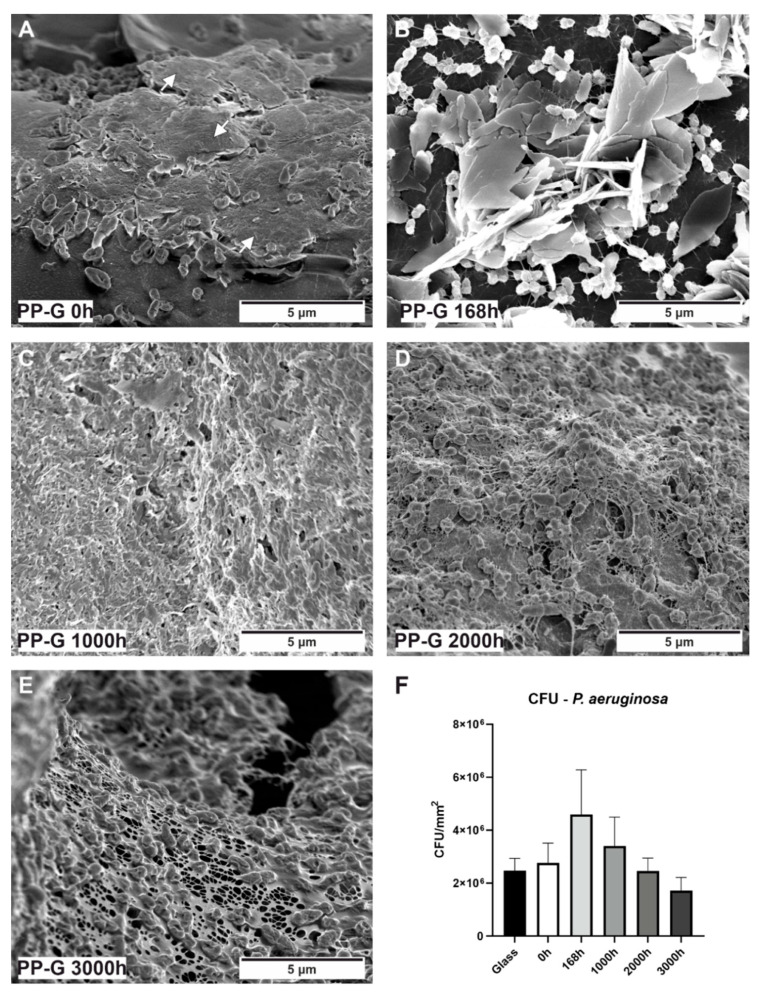
Influence of aged polypropylene samples on bacterial attachment. SEM image of (**A**) *P. aeruginosa* attached to PP-G after 0 h with EPS indicated by white arrows, (**B**) after 168 h with visible EBS, (**C**) after 1000 h, (**D**) after 2000 h, and (**E**) after 3000 h. (**F**) CFU assay results quantifying the attached *P. aeruginosa*, compared to the reference glass sample. Mean ± standard error of the mean are shown.

To gain insight into bacterial attachment in terms of biofilm formation, all PP samples were incubated for 24 h in liquid LB-medium with a wild-type *P. aeruginosa* isolate (Figure 4 and Figure 5). A standard glass slide was used as a reference material. SEM observations revealed that *P. aeruginosa* is able to attach on all tested unaged PP samples (Figure 4B,C and Figure 5A). Even the first stages of biofilm formation are visible, such as three-dimensional growth (Figure 4B,C) and the formation of an extracellular polymeric substance (EPS), visible as a “slime”-like continuous film covering the bacteria (Figure 5A white arrows). To corroborate these visual results, the CFU assay was performed as a direct quantification method of viable colonies attached to the surfaces. PP-T and PP-G show a similar number of viable colonies of about 2 × 10^6^ colonies per mm^2^, compared to the reference glass material. Interestingly, approximately 2.5 times more live colonies could be detected on the unfilled PP material (around 5 × 10^6^ colonies per mm^2^). Note that among the unaged (0 h) samples, only on the PP sample EBS was present.

The influence of PP-aging in the detergent solution on bacterial attachment was tested exemplarily for PP-G. The bacterial testing was conducted on the samples after the aging process was performed. On all aged samples bacterial attachment and network structures are visible (Figure 5B–E). After 168 h, crystalline EBS is evident on the surface, with bacteria networks inside (Figure 5B). A section was chosen where the crystalline EBS structure is not covered by surface overgrowing bacteria. The quantification of viable colonies revealed that there is a higher tendency of attachment to the 168 h aged PP-G of about 4.5 × 10^6^ colonies per mm^2^ (Figure 5F). There is a decreasing tendency of attachment with ongoing aging observable. The 168 h aged PP-G sample exhibited the highest amount of EBS on the surface according to the microscopy results.

**Figure 6:**
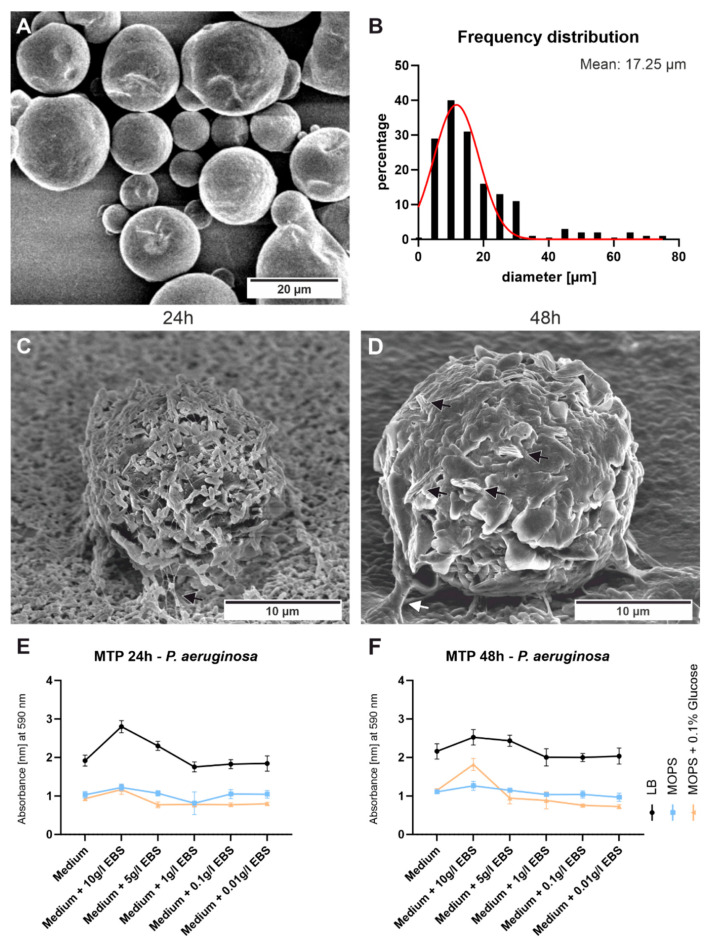
Observation of the influence of pure EBS on *P. aeruginosa* attachment and biofilm formation. (**A**) SEM image of pure EBS micropearls, (**B**) frequency diameter distribution of EBS particles with Gaussian fit central peak at 17.25 µm. (**C**) *P. aeruginosa* attachment to a glass slide in LB medium supplemented with 1 g/Lpure EBS, after an incubation period of 24 h, *P. aeruginosa* forms filament-like network structures (black arrow) and (**D**) after 48 h, white arrow pointing on the EPS layer, black arrows pointing on the crystalline EBS fragments protruding from the particle. Results of microtiter plate method with crystal violet staining, after an incubation period of (**E**) 24 h and (**F**) 48 h. Mean ± standard deviation are shown.

In addition, the influence of pure EBS on the attachment as well as the formation of biofilms of *P. aeruginosa* was tested. The SEM image of pure EBS (Figure 6A) shows that it is present in micro-pearled particles. A size measurement revealed that most of the particles have a diameter of about 17 µm (Figure 6B). For the initial bacterial tests, LB medium was supplemented with 1 g/L EBS in powder form. In a 6-well plate, in which a glass slide was placed, the incubation was performed for 24 and 48 h with the presence of *P. aeruginosa*. After an incubation period of 24 h, *P. aeruginosa* forms filament-like network structures (Figure 6C black arrow). EBS particles embedded and completely covered by *P. aeruginosa* are visible. After 48 h, biofilm formation proceeds, as shown by horizontal growth and slime-like structures (Figure 6D). Bacterial filamentous extensions (network structures) visible on the EBS particles after 24 h are now covered by an extracellular polymeric substance (EPS) layer which is recognizable as “slime” or “mucus” (Figure 6D white arrow). EBS particles are completely covered in mucus and are apparently kept on the surface by it. The crystalline, edged EBS fragments, protruding from the round particle, especially after 48 h of incubation, suggest a transition into the thermodynamic stable crystalline form, such as on the aged samples (Figure 6D black arrows). A penetrating biofilm is visible between the crystalline EBS structures, which could indicate biodegradation on the micro-pearled particles.

To quantify the influence of EBS on biofilm formation the microtiter plate assay was performed. LB medium was supplemented with different concentrations of EBS and filled into a 96-well plate. *P. aeruginosa* was then inoculated followed by an incubation period of 24 and 48 h. In addition, the minimal medium MOPS (supplemented with 1% glucose and without) was used to draw better conclusions about whether *P. aeruginosa* can use EBS as a carbon source. After the medium was discarded followed by washing, the remaining biofilm was stained with crystal violet. By measuring the optical density, conclusions can be drawn about the amount of formed biofilm. To ensure that the observed effect was not caused by EBS residues on the plate, a negative plate without *P. aeruginosa* was performed for each condition. These plates were also subsequently stained with crystal violet. These values were then subtracted from the values obtained in the presence of *P. aeruginosa*. After 24 h, most biofilm residue could be measured in the presence of 10 g/L EBS for all tested media (Figure 6E). The most biofilm residue is noticeable in LB Medium. In MOPS Medium, small increases can be seen for both variants. After an incubation period of 48 h, highest optical density can be measured again for the presence of 10 g/L EBS (Figure 6F). The LB medium with 5 g/L EBS also revealed a higher optical density compared to the control. Especially for MOPS medium supplemented with 10 g/L EBS higher values compared to the control can be observed.

## 4. Discussion

With a rising worldwide demand of polypropylene (77.91 million metric tons in 2022) gaining insights into the aging process is indispensable [27]. Polypropylene is a semi-crystalline thermoplastic polymer with excellent chemical stability and a variety of applications [14]. Aging in liquid media does not only include the material degradation but also biological processes, like bacterial contamination, must be considered. To the best of our knowledge there are no studies investigating the aging of polypropylene in detergent solutions. Here we present the first results of surface aging of industrial used polypropylene.

In thermal and photo oxidation, there are two main signs for degradation: surface cracking and hardening [28,29,30,31]. For aging in a detergent the same effects apply, but blooming can be more pronounced. How robust a polypropylene sample is against aging processes depends on molecular parameters, the involved additives, and the environment to which it is exposed to. Some additives of the analysed industrial samples are unknown, which complicates the comparability. Nevertheless, the results show how long industrial polypropylene remains functional in a detergent solution at 95 °C and how the surface structure changes, setting a benchmark for further research. Polypropylene samples with a proper additive package will last up to 4000 h before cracking occurs. Using talc as a filler has its benefits like achieving a higher thermal conductivity and especially improving the rigidity [32,33]. Particularly with regard to construction is the application of talc important enabling less shrinkage and warpage [34,35]. In terms of durability, a talc filling has a negative impact as presented here by the early crack formation on the surface during the aging process. The AFM measurements revealed that the formed fibre-like EBS on the affected samples may act as a sacrificial layer. These samples still possessed the initial surface morphology of the untreated samples after 4000 h in the detergent at 95 °C. The PP sample lost the initial injection moulding skin during aging, indicating a higher material removal of the polypropylene surface without the EBS fibrous layer on the surface.

The main issue that should be addressed here is the use of EBS in polypropylene manufacturing with regard to polymer aging. First, the results show that the EBS does not behave similarly in different polypropylene matrixes. To function properly as lubricant, meaning to reduce the friction between two surfaces, it is indispensable to be located on the surface. In two of the three studied industrial samples that was not the case. The EBS did not appear on the surface of the untreated as received samples but was solved in the polypropylene bulk. Browning et al. observed the same behaviour for erucamide in talc filled polypropylene [36]. Erucamide is a more frequently used lubricant than EBS. They claim that the polar amide end group of the erucamide can interact with untreated talc and thereby prevent it from diffusing to the surface [36]. The EBS was not detectable on the surface of the unaged talc filled sample PP-T and the unaged glass fibre sample PP-G. Contrary to the erucamide, the EBS does not have a polar end group and glass fibres are typically treated with silane to become nonpolar in order to incorporate the fibres in the polypropylene matrix. Therefore, it is unlikely that the EBS is trapped in the bulk due to polar interactions with the filling material. Blooming mainly depends on the solubility and diffusion constant of an additive in the polymer. If the solubility of an additive is too low, it undergoes a phase separation and migrates to the surface. Among other things, the solubility of an additive depends on the crystallinity of the polymer. In general, additives are dissolved in the amorphous phase but not in the crystalline phase [37]. The DSC measurements revealed that the PP-T and PP-G sample exhibited a much lower crystallinity than the PP sample. Therefore, we argue that the less compatible PP sample already exuded most of the EBS during the manufacturing process. Thus, we can detect the EBS on the surface of the PP sample in the 0 h FTIR spectrum. This is intended for lubricants because it ensures that the lubricant is located on the surface to reduce friction. Contrary to the PP sample, we could not detect EBS on the 0 h PP-T and PP-G sample. Here the EBS did not exude from the polymer matrix during manufacturing because no EBS bands can be detected in the 0 h spectra of PP-T and PP-G. The heating in liquid environment enables the EBS to diffuse to the surface. Since the less compatible PP sample already exuded most of the EBS during the manufacturing process, there are no or very little remains left in the bulk which can diffuse to the surface during the aging process. In the more compatible samples PP-T and PP-G, there is still EBS dissolved in the bulk, so that it can diffuse to surface and form the observed fibre-like crystals.

Interestingly, antioxidants form similar fibrous structures on the surface of polyurethane after blooming [38,39,40]. Thus, the appearance of fibre-like crystalline structures is a strong indication for antioxidants or EBS on the surface which can easily be distinguished using ATR-FTIR. Antioxidants were not detectable on the here investigated samples and therefore antioxidant blooming can be excluded. This is a beneficial result for polypropylene aging in heated solutions in general, because bloomed antioxidants result in a lower protection efficiency against oxidation [41].

There are numerous applications in medicine, household, food, or automotive industry where polypropylene is used in a heated liquid environment [14,15]. Nevertheless, the microbial load of such materials, especially in medical use, should not be neglected. Polypropylene meshes are widely used for urological treatments like inguinal hernia surgery [42], stress urinary incontinence [43], or pelvic floor implants [44]. However, biofilm formation can also occur on such materials, which can cause serious health risks, including sepsis [45,46,47]. Due to an increasing environmental awareness, washing at low temperatures is often considered to safe resources [48,49]. However, this can also increase the microbial load in terms of biofilms in household appliances [11,50]. Additionally, the surface morphology changes in PP induced by aging are important in the context of microbial contamination because uneven or cracked surfaces hinder the effectiveness of disinfection methods [51].

The here tested industrial PP samples revealed a surface covering *P. aeruginosa* attachment and even first stages of biofilm formation. Among the unaged samples the highest number of viable colonies could be observed on the PP sample. Since PP was the only unaged sample with EBS on the surface, this indicates that the EBS is likely to enhance the bacterial attachment. Interestingly, initial biofilm stages could be observed on the PP-G sample. This might be a stressed-induced growth reaction of *P. aeruginosa* to the glass fibres or the silane on the surface.

The influence of aging on bacterial adhesion in the form of biofilm formation was tested exemplary for sample PP-G. Surface covering growth could be observed for all aging timesteps. The highest number of viable colonies could be found after crystalline EBS structures appeared on the surface. This underlines the enhanced bacterial attachment and biofilm formation in the presence of EBS. To our knowledge, the effect of pure EBS in terms of bacterial growth and biofilm formation has not yet been studied. Saunier et al. presented the elevated bacterial growth of S. aureus in the presence of antioxidants [40]. They conclude that these effects are due to a more adhesive surface. This could also be the case for the observed EBS crystalline structures. First conclusions of EBS promoting biofilm formation of *P. aeruginosa* can be drawn by the observed effect visible after an incubation period of 48 h in LB medium supplemented with 1 g/L EBS. SEM observations clearly revealed that in comparison to a 24 h period, enhanced biofilm formation around the EBS particles appeared. The visible crystalline fragments occurred on the micro-pearled EBS like on the aged PP samples. The penetration of the biofilm, visible on the micro-pearls, might indicate a degradation of EBS by *P. aeruginosa*. To test whether EBS can also be used as a nutrient source by *P. aeruginosa*, a microtiter plate assay was additionally performed. It is clearly visible that EBS promotes biofilm formation of *P. aeruginosa* when used in higher concentrations. Even in the minimal medium without an additional carbon source, a slight increase in optical density is observed at high concentrations of EBS, which indicates increased biofilm growth. Suggesting that an additional carbon source, for example glucose, enhances the positive effect of EBS.

## 5. Conclusions

In this study, it was shown that the here tested industrial samples can withstand up to 4000 h of aging in a detergent at 95 °C. Using talc as filler seems to be disadvantageous for the longevity because it increases the probability of early crack formation. Nevertheless, talc is needed for its higher Young’s modulus as well as less shrinkage and warpage in construction. The results revealed that the use of EBS in polypropylene can change the surface morphology and properties drastically during liquid aging. On the one hand, it may act beneficially as a sacrificial layer against the aging processes. On the other hand, biofilm formation by *P. aeruginosa* is found to be enhanced when EBS structures are present on the surface of all polypropylene samples. EBS itself benefits biofilm formation even when it represents the only carbon source. The underlying effects should be subject to further research.

The use of EBS and similar slip agents in industrial PP, especially in long-term use, needs to be better understood and applied, particularly with regard to surface modification caused by blooming and microbial contamination.

## Data Availability

The data presented in this study are available from the corresponding authors on request.

## Supplementary Materials

The following supporting information can be downloaded at: https://www.mdpi.com/article/10.3390/polym15051247/s1, Figure S1: AFM height image of PP-G after 110 h in detergent at 95 °C. Areas where the polypropylene surface is not yet covered by EBS are still present. The height profile taken along the white line shows a maximum height of 2 µm, serving as an estimate for the thickness of the resulting EBS layer; Figure S2: Dimensions of the injection moulded polypropylene test specimens type A1. Image taken from ISO 20753:2008(E); Figure S3: AFM images of the unaged samples (A) PP, (B) PP-T und (C) PP-G showing the injection moulding skin characterised by the parallel grooves. Black arrows indicate the injection direction; Figure S4: SEM images of a vortexed PP sample present the detachment of the biofilm, since no bacteria are present on the surface, only residues formed due to previous adhesion are visible.
