# Supplementary material for: Aging of Industrial Polypropylene Surfaces in Detergent Solution and Its Consequences for Biofilm Formation
Source: Polymers (Basel). 2023 Feb 28;15(5):1247. doi: 10.3390/polym15051247 (PMC10006934; doi:10.3390/polym15051247)
Supplement: Supplementary file 1 [file polymers-15-01247-s001.zip › polymers-2253645-supplementary.pdf]

polymers

MDPI

Supplementary Materials:

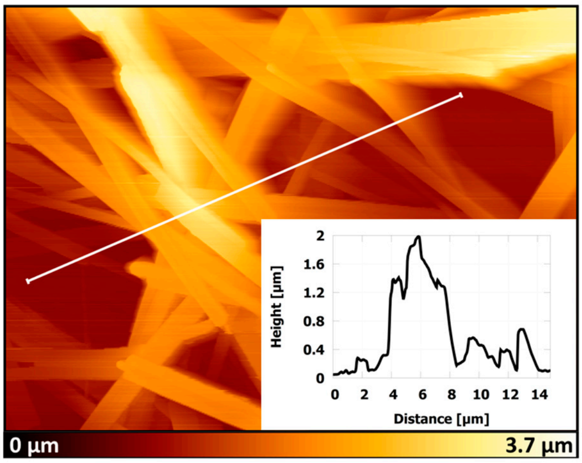

Figure S1. AFM height image of PP-G after 110 h in detergent at 95°C. Areas where the polypropylene surface is not yet covered by EBS are still present. The height profile taken along the white line shows a maximum height of 2 µm, serving as an estimate for the thickness of the resulting EBS layer.

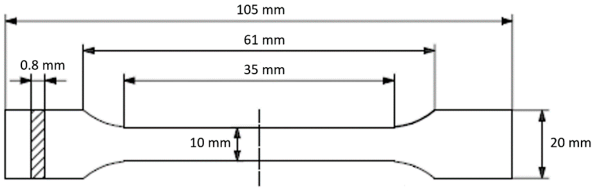

Figure S2. Dimensions of the injection moulded polypropylene test specimens type A1. Image taken from ISO 20753:2008(E).

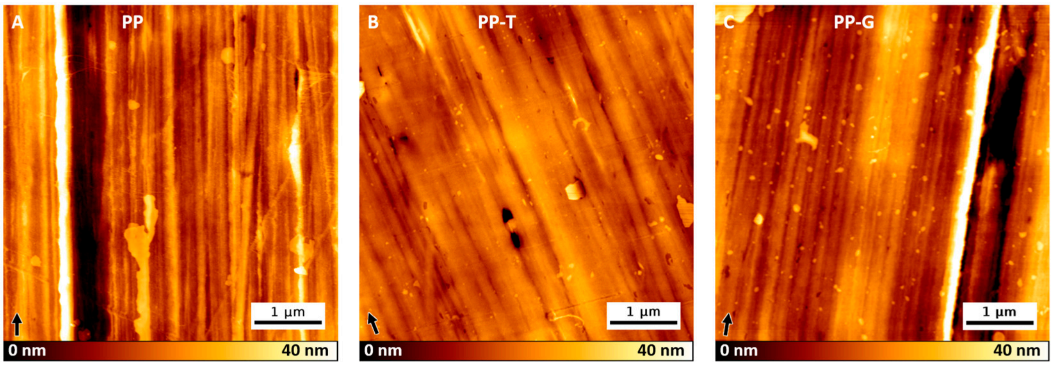

Figure S3. AFM images of the unaged samples (A) PP, (B) PP-T und (C) PP-G showing the injection moulding skin characterised by the parallel grooves. Black arrows indicate the injection direction.

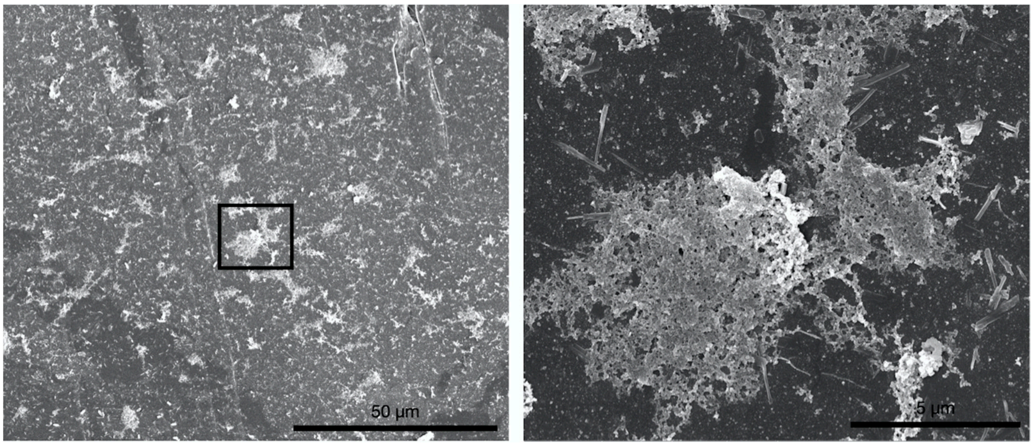

Figure S4. SEM images of a vortexed PP sample present the detachment of the biofilm, since no bacteria are present on the surface, only residues formed due to previous adhesion are visible.
